# Effects of *Ecklonia cava* Extract on Neuronal Damage and Apoptosis in PC-12 Cells against Oxidative Stress

**DOI:** 10.4014/jmb.2012.12013

**Published:** 2021-03-26

**Authors:** Yong Sub Shin, Kwan Joong Kim, Hyein Park, Mi-Gi Lee, Sueungmok Cho, Soo-Im Choi, Ho Jin Heo, Dae-Ok Kim, Gun-Hee Kim

**Affiliations:** 1 Graduate School of Biotechnology, Kyung Hee University, Yongin 17104, Republic of Korea; 2 Department of Applied Biotechnology, Ajou University, Suwon 16499, Republic of Korea; 3 Bio-Center, Gyeonggido Business and Science Accelerator, Suwon 16229, Republic of Korea; 4 Department of Food Science and Technology, Pukyong National University, Busan 48513, Republic of Korea; 5 Department of Foods and Nutrition, Duksung Women's University, Seoul 01369, Republic of Korea; 6 Division of Applied Life Science (BK21), Institute of Agriculture and Life Science, Gyeongsang National University, Jinju 52828, Republic of Korea; 7 Department of Food Science and Biotechnology, Kyung Hee University, Yongin 17104, Republic of Korea

**Keywords:** Dieckol, *Ecklonia cava*, oxidative stress, phlorotannin-rich extract, seaweed

## Abstract

Marine algae (seaweed) encompass numerous groups of multicellular organisms with various shapes, sizes, and colors, and serve as important sources of natural bioactive substances. The brown alga *Ecklonia cava* Kjellman, an edible seaweed, contains many bioactives such as phlorotannins and fucoidans. Here, we evaluated the antioxidative, neuroprotective, and anti-apoptotic effects of *E. cava* extract (ECE), *E. cava* phlorotannin-rich extract (ECPE), and the phlorotannin dieckol on neuronal PC-12 cells. The antioxidant capacities of ECPE and ECE were 1,711.5 and 1,050.4 mg vitamin C equivalents/g in the ABTS assay and 704.0 and 474.6 mg vitamin C equivalents/g in the DPPH assay, respectively. The dieckol content of ECPE (58.99 mg/g) was approximately 60% higher than that of ECE (36.97 mg/g). Treatment of PC-12 cells with ECPE and ECE increased cell viability in a dose-dependent manner. Intracellular oxidative stress in PC-12 cells due to ECPE and ECE decreased dose-independently by up to 63% and 47%, respectively, compared with the stress control (323%). ECPE reduced the production of the pro-apoptotic proteins Bax and caspase-3 more effectively than ECE. Early and late apoptosis in PC-12 cells were more effectively decreased by ECPE than ECE treatments. From the results obtained in this study, we concluded that ECPE, which is rich in phlorotannins, including the marker compound dieckol, may be applied to the development of functional materials for improving cognition and memory.

## Introduction

Neurodegenerative diseases, such as Alzheimer’s disease (AD) and Parkinson’s disease (PD), are caused by neuronal cell death, protein misfolding, and aggregation in neuronal cells [1]. A common cause of neurodegeneration is oxidative stress, which results in nerve cell dysfunction or death [2]. Reactive oxygen species (ROS) are a byproduct of cellular metabolism in the body, and excessive amounts can injure human tissue and, ultimately, induce neurodegeneration [3]. The brain consumes large amounts of oxygen during energy production and because brain tissue has relatively low antioxidant levels and a high polyunsaturated fatty acid content, it is susceptible to oxidative damage induced by the overproduction of ROS.

The brain has the highest energy consumption among organs of the body, accounting for up to 20-25% of the body’s energy reserves during oxygen metabolism [4, 5]. Oxidative phosphorylation of mitochondria in the cell body, axons, and neuronal synapses results in the production of ATP [6]. Electron leaks during mitochondrial metabolism cause oxidative stress and produce ROS such as superoxide anion, which affect nuclear DNA (nuDNA) and mitochondrial DNA (mtDNA) [7]. The mitochondria undergo membrane expansion after exposure to oxidative stress and their expansion and collapse induce Bcl-2-associated X protein (Bax)-mediated apoptosis and, consequently, caspase- and cytochrome c-mediated apoptosis [8]. mtDNA has a higher mutation rate and less effective recovery mechanisms than nuDNA [9]. As the body ages, mtDNA damage and mitochondrial malfunction from oxidative stress increase, escalating the possibility of neurodegenerative diseases.

Seaweeds abundant in secondary metabolites are considered not only excellent sources of food but also therapeutic material [10, 11]. Numerous studies have been conducted on the various bioactive compounds in seaweeds to evaluate their antioxidant and neuroprotective effects [12-14]. Many of these bioactive compounds are thought to be produced by the algae for self-protection, adaptation to harsh habitats, and in response to nutritional deficiencies, high heat, high salinity, and low-light intensities [15]. As good natural sources of antioxidants, marine algae have been used in many studies to find bioactives beneficial to health [10, 11, 16].

*Ecklonia cava* Kjellman (class Phaeophyceae; family Laminariaceae) is an edible, perennial brown alga that is widely distributed in rocky areas 5-25 m below sea level in coastal areas of Korea and Japan [16]. A variety of bioactive compounds are produced by *E. cava*, including phlorotannins, sulfated polysaccharides (i.e., fucoidans), peptides, and carotenoids [16]. Phlorotannins are polymers of phloroglucinol (1,3,5-trihydroxybenzene) and a class of major bioactive polyphenolic compounds found in this alga, which suggests that it is a good source of natural antioxidants with potential applications in preventing the oxidative insult of neurons. By comparison with other seaweeds, the major phlorotannins in *E. cava* include eckol, eckstolonol, phlorofucofuroeckol A, 7-phloroeckol, dieckol, 6,6′-bieckol, 8,8′-bieckol, and triphlorethol A [17, 18]. Phlorotannins from marine algae also show anti-proliferative, antihypertensive, anti-inflammatory, and anti-diabetic properties [11, 17, 19].

Previous studies have shown that the antioxidant capacity of *E. cava* extract is greater than that of other algae [20]. The fucoidan extract of *E. cava* has neuroprotective effects against oxidative stress that act through the downregulation of mitochondrial-mediated proteins, such as Bax [12]. Therefore, the purpose of this study was to investigate the antioxidant and neuroprotective effects of *E. cava* extract (ECE), *E. cava* phlorotannin-rich extract (ECPE), and a phlorotannin dieckol on hydrogen peroxide-induced oxidative stress in neuronal PC-12 cells.

## Materials and Methods

### Reagents

We purchased 2,2-diphenyl-1-picrylhydrazyl (DPPH), ascorbic acid, 2,2′-azino-bis(3-ethylbenzothiazoline-6-sulfonic acid) diammonium salt (ABTS), 3-(4,5-dimethyl-2-thiazolyl)-2,5-diphenyl-2H-tetrazolium bromide (MTT), dimethyl sulfoxide (DMSO), hydrogen peroxide, 2′,7′-dichlorofluorescin diacetate (DCFH-DA), and Dulbecco’s phosphate-buffered saline (DPBS) from Sigma-Aldrich Co., LLC (USA). Fetal bovine serum (FBS) was purchased from Gibco Thermo Scientific Inc. (USA). Hanks’ balanced salt solution (HBSS), Roswell Park Memorial Institute (RPMI)-1640, trypsin-EDTA, and penicillin-streptomycin solutions were purchased from Welgene Inc. (Republic of Korea). Laemmli sample buffer was obtained from FUJIFILM Wako Pure Chemical Corp. (Japan). RIPA Lysis and Extraction Buffer and Pierce bicinchoninic acid (BCA) Protein Assay Kit was purchased from Thermo Fisher Scientific Inc. Primary and secondary antibodies were obtained from Cell Signaling Technology (USA). All reagents and chemicals were of analytical or high-performance liquid chromatography (HPLC) grade unless otherwise specified.

### Preparation of ECE and ECPE

The *E. cava* specimens used in this study were collected from Aewol-eup, Jeju Island, Republic of Korea. Fresh *E. cava* was washed with water and dried at 65°C. Then, the dry seaweed was immersed in 80% (v/v) aqueous ethanol for 24 h to prepare the ECE, while the ECPE was prepared by extracting dry *E. cava* at 60°C for 6 h with 10 times the 70% (v/v) aqueous ethanol. The ECE and ECPE were filtered through Whatman No. 2 filter paper (Whatman International Ltd., UK), concentrated, and lyophilized.

### Determination of Antioxidant Capacity

Antioxidant capacity was measured using the ABTS and DPPH radicals. The antioxidant capacity was expressed as milligrams of vitamin C equivalents (VCE)/g of dried weight. In the ABTS radical scavenging assay [21], the ABTS radical solution was adjusted to an absorbance of 0.650 ± 0.020 at 734 nm. The reactions between the ABTS radicals and the ECE, ECPE, or dieckol took place at 37°C for 10 min. We measured the decrease in absorbance of the resulting solution at 734 nm using a SPECTRONIC 200 spectrophotometer (Thermo Fisher Scientific Inc.).

In the DPPH radical scavenging assay [21], DPPH radicals (0.1 mM) were dissolved in 80% (v/v) aqueous methanol, and the absorbance for the DPPH radicals was set to 0.650 ± 0.020 at 517 nm. The reactions between the DPPH radicals and the ECE, ECPE, or dieckol were allowed to proceed at 23°C for 30 min. The decrease in absorbance of the resulting solution was monitored at 517 nm using a SPECTRONIC 200 spectrophotometer.

### Quantification of Dieckol in ECE and ECPE Using Reversed-Phase HPLC

The concentrations of dieckol in the ECE and ECPE were quantitatively analyzed using an HPLC system (Alliance 2695; Waters Corp., USA) equipped with a UV/visible light detector (2489; Waters Corp.) and a ProntoSIL 120-5-C18 ace-EPS (250 × 4.6 mm, 5 μm; Bischoff Chromatography, Germany). Gradient elution was carried out with water (solvent A) and acetonitrile (solvent B). The gradient conditions were as follows: 80% A/ 20% B at 0 min, 60% A/40% B at 20 min, 20% A/80% B at 30 min, 80% A/20% B at 40 min, and 80% A/20% B at 45 min. The flow rate of the mobile phase was 1.0 ml/min, and the injection volume was 5 μl. The temperature of the analytical column was maintained at 30°C. Dieckol was quantified using a standard curve for authentic standard dieckol.

### Cell Culture

PC-12 cells were used to examine the neuroprotective effects of hydrogen peroxide-induced oxidative stress. The PC-12 cell line, which is derived from a transplantable rat pheochromocytoma, was purchased from American Type Culture Collection (USA). The PC-12 cells were cultured in RPMI-1640 medium containing 10%heat-inactivated FBS, 100 units/ml penicillin, 0.25 μg/ml Fungizone (amphotericin B), and 100 μg/ml streptomycin. Neuronal PC-12 cells were cultured in a humidified incubator (Forma 3110 CO_2_ Incubator; Thermo Fisher Scientific Inc.) with 5% CO_2_ at 37°C.

### Cytotoxicity and Viability of PC-12 Cells

The effects of the ECE, ECPE, and dieckol on the cytotoxicity and cell viability of the PC-12 cell line were estimated using the MTT formazan reduction assay [22]. The PC-12 cells were precultured for 24 h at counts of 2×10^4^ cells/well in 96-well, round, clear plates. After preculturing, the culture medium was removed, and the PC-12 cells were treated with FBS-free medium containing ECE, ECPE, or dieckol at 10, 25, and 50 μg/ml for 24 h. The cells were treated with 200 μM H_2_O_2_ for 1 h, and then with 0.5 mg/ml MTT for 3 h. The resulting MTT formazan products generated by the reduction reactions in the PC-12 cells were dissolved by adding DMSO. The amount of MTT formazan dissolved in DMSO was determined by measuring the absorbance at 570 nm in a microplate reader (Synergy HTX; BioTek Instruments, Inc., USA). The cytotoxicity and viability of the PC-12 cells were expressed as the percentage (%) of viable cells relative to untreated control cells (100%).

### Determination of Intracellular Oxidative Stress Levels in PC-12 Cells

The intracellular oxidative stress levels of the neuronal PC-12 cells were evaluated using the fluorescent probe DCFH-DA [23]. PC-12 cells at 2 × 10^4^ cells/well were precultured in 96-well, round, clear plates for 24 h. Then, the cells were treated with ECE, ECPE, or dieckol at 10, 25, and 50 μg/ml for 24 h. After removing the sample-containing medium, the PC-12 cells were incubated with 50 μM of DCFH-DA in HBSS for 1 h, then treated with 100 μM of H_2_O_2_ in HBSS for 1 h. Fluorescence was measured at 485 nm (detection wavelength) and 535 nm (emission wavelength) using a microplate reader (Synergy HTX; BioTek Instruments, Inc.). The intracellular oxidative stress levels of the PC-12 cells were expressed as the percentage (%) of fluorescence intensity compared with the untreated control cells (100%).

### Analysis of PC-12 Cell Apoptosis Using Flow Cytometry

Navios EX flow cytometry (Beckman Coulter Life Sciences, USA) was used to observe apoptosis of the PC-12 cells, and dot plots were obtained using Kaluza 1.1 software (Beckman Coulter Life Sciences). The PC-12 cells were precultured for 24 h at 1 × 10^6^ cells/well in 6-well, round, clear plates and then treated with ECE, ECPE, or dieckol at 25 and 50 μg/ml for 24 h. After removing the medium with the sample, the cells were washed with DPBS. Then, PC-12 cell apoptosis was induced by adding 200 μM H_2_O_2_ for 1 h. The cells were washed and detached using PBS, resuspended in the binding buffer, and stained with annexin V-fluorescein isothiocyanate (FITC)/propidium iodide (PI) staining solution for 15 min. The PC-12 cells were quantitated using a flow cytometry system, and at least 10,000 events were analyzed and recorded. Apoptotic PC-12 cells were expressed as a percentage (%) of the total cell number.

### Western Blot Analysis

The PC-12 cells at 1 × 10^6^ cells/well were precultured for 24 h in 6-well, round, clear plates. They were then treated with 25 or 50 μg/ml of ECE, ECPE, or dieckol for 24 h. The sample-containing medium was removed, and the PC-12 cells were washed and lysed in RIPA buffer for 15 min on ice. Completely lysed cells were centrifuged at 13,000 ×*g* for 30 min, and the protein content of the supernatant was quantified by BCA assay. Lysed cell samples with the same protein were added to Laemmli sample buffer and denatured at 100°C for 8 min. Proteins that reacted with the Laemmli sample buffer were used for 4-20% sodium dodecyl sulfate-polyacrylamide precast gel electrophoresis, then transferred to polyvinylidene difluoride membranes. The membranes were blocked using 2.5% bovine serum albumin in Tris-buffered saline with Tween 20 for 1 h and then immunoblotted overnight at 4°C with primary antibodies to Bax, B-cell lymphoma 2 (Bcl-2), caspase-3, and glyceraldehyde-3-phosphate dehydrogenase (GAPDH). The membranes immunoblotted with the primary antibodies were incubated with the corresponding secondary antibodies for 60 min at an ambient temperature. We then visualized the membrane bands with an LAS-4000 (Fujifilm Corp., Japan). The developed image showing the bands for caspase-3, Bax, and Bcl-2 proteins was densitometrically analyzed using Image J software (National Institutes of Health, USA). The western blot results were reported as the ratios of caspase-3/GAPDH, Bax/GAPDH, and Bax/Bcl-2.

### Statistical Analysis

All experiments were performed three times. The results are expressed as the mean ± SD. The statistical significance of the differences between the test groups was analyzed for comparison using SPSS version 22 (SPSS Science, USA). Statistical relevance was determined using analysis of variance, and significant differences were verified by Duncan’s multiple range test at the *p* < 0.05 confidence level.

## Results

### Antioxidant Capacity

Antioxidant capacities were evaluated using the ABTS and DPPH radical scavenging assays. As shown in Table 1, the antioxidant capacity of ECPE was higher than that of ECE in both the ABTS and DPPH assays. The antioxidant capacities of ECE, ECPE, and dieckol were 1,050.4; 1,711.5; and 2,328.3 mg VCE/g in the ABTS assay and 474. 6, 704.0, and 910.5 mg VCE/g in the DPPH assay, respectively.

### Quantification of Dieckol Using Reversed-Phase HPLC

**Fig. 1:**
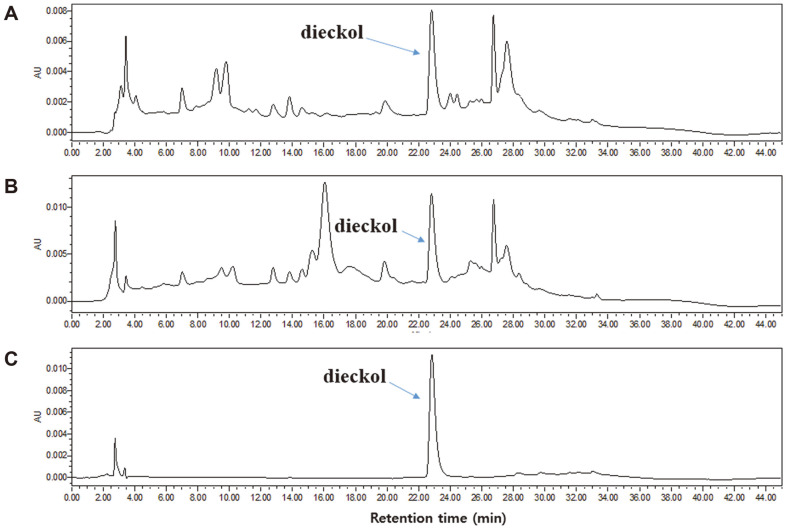
HPLC traces (230 nm) of (A) *E. cava* extract, (B) *E. cava* phlorotannin-rich extract, and (C) dieckol.

The chromatographic conditions were optimized to obtain the HPLC analysis of ECE and ECPE (Fig. 1). Dieckol was eluted at approximately 23.0 min (Fig. 1), and ECE and ECPE contained 36.97 mg/g and 58.99 mg/g of dieckol, respectively (Table 2). ECPE was found to have an approximately 1.6-fold higher amount of dieckol than ECE.

### Effects of ECE, ECPE, and Dieckol on PC-12 Cell Viability

**Fig. 2:**
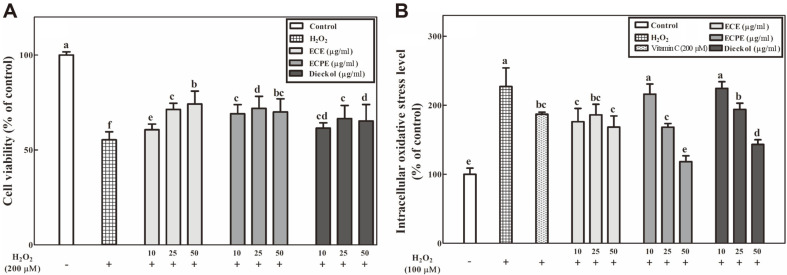
Neuroprotective effects of *E. cava* extract (ECE), *E. cava* phlorotannin-rich extract (ECPE), and dieckol on (A) cell viability and (B) intracellular oxidative stress in neuronal PC-12 cells under oxidative stress. The viability of PC-12 cells under oxidative stress induced by H_2_O_2_ was measured using the MTT assay. Intracellular oxidative stress in PC-12 cells under oxidative stress induced by H_2_O_2_ was measured using the DCFH-DA assay. Data are displayed as the mean ± SD (bars) of three replicates. Different letters indicated a statistical difference at the *p* < 0.05 level among data groups according to Duncan’s multiple range test.

The cytotoxic effects of ECE, ECPE, and dieckol on PC-12 cells were assessed using the MTT assay. Concentrations of ECE, ECPE, and dieckol associated with a cell viability equal to or greater than 90% were considered non-toxic. ECE, ECPE, and dieckol were found to be non-toxic up to 50 μg/ml (data not shown). PC-12 cells treated with H_2_O_2_ (200 μM) showed a 44.6% decrease in cell viability compared with the untreated control cells (100%) (Fig. 2A). As shown in Fig. 2A, treatment with ECE, ECPE, or dieckol increased the viability of PC-12 cells subjected to H_2_O_2_-induced oxidative stress compared with the negative control (H_2_O_2_ group).

### Effects of ECE, ECPE, and Dieckol on Intracellular Oxidative Stress Levels of PC-12 Cells

The intracellular oxidative stress levels of PC-12 cells were measured using the DCFH-DA assay. The negative control cells, which were treated with 200 μM H_2_O_2_, had approximately 227.1% of the intracellular oxidative stress levels observed in the untreated control group (100%), while treatment of the positive control group with vitamin C (200 μM) showed approximately 187.0% intracellular oxidative stress levels compared with the untreated control group (100%) (Fig. 2B). ECE, ECPE, and dieckol reduced the intracellular oxidative stress levels of the PC-12 cells, as much as or more than, the vitamin C treatment (Fig. 2B). The PC-12 cells in the ECPE and dieckol groups showed a concentration-dependent decrease in intracellular oxidative stress levels. Treatment of PC-12 cells with 50 μg/ml ECPE reduced the intracellular oxidative stress to levels similar to those in the control group (Fig. 2B).

### Effects of ECE, ECPE, and Dieckol on Apoptosis of PC-12 Cells

**Fig. 3:**
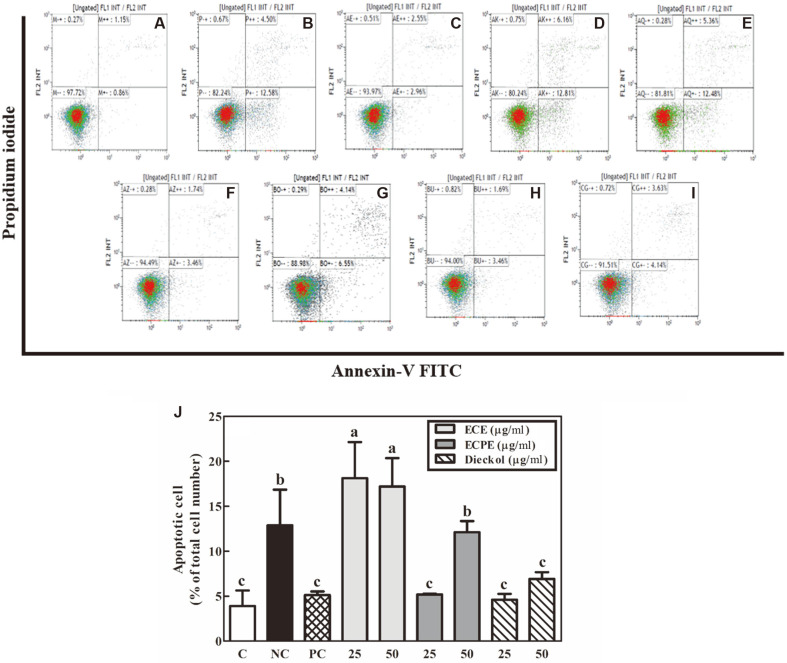
Effects of *E. cava* extract (ECE), *E. cava* phlorotannin-rich extract (ECPE), and dieckol on apoptosis of neuronal PC-12 cells under oxidative stress (200 μM H_2_O_2_) using flow cytometry analysis. Dot plots for flow cytometry analysis of apoptosis: (**A**) control, (**B**) H_2_O_2_ (200 μM), (**C**) H_2_O_2_ (200 μM) + vitamin C (200 μM), (**D**) H_2_O_2_ (200 μM) + ECE (25 μg/ml), (**E**) H_2_O_2_ (200 μM) + ECE (50 μg/ml), (**F**) H_2_O_2_ (200 μM) + ECPE (25 μg/ml), (**G**) H_2_O_2_ (200 μM) + ECPE (50 μg/ml), (**H**) H_2_O_2_ (200 μM) + dieckol (25 μg/ml), and (**I**) H_2_O_2_ (200 μM) + dieckol (50 μg/ml). (**J**) Graph quantifying the percentage (%) of apoptotic PC-12 cells. C, control; NC, negative control (200 μM H_2_O_2_); PC, positive control (200 μM vitamin C). Data are displayed as the mean ± SD (bars) of three replicates. Different letters indicated a statistical difference at the *p* < 0.05 level among data groups according to Duncan’s multiple range test.

The flow cytometry results for the quantitative analysis of the apoptotic cells are shown in Fig. 3. Dot plots of the flow cytometry results showing apoptosis of PC-12 cells treated with vitamin C, ECE, ECPE, and dieckol are shown in Figs. 3A-3I. The apoptotic PC-12 cells of the untreated control, negative control, and positive control (200 μM vitamin C) groups were 3.90%, 12.88%, and 5.13% of the total cell number, respectively. Treatment of PC-12 cells with ECPE or dieckol decreased the apoptotic cells (5.18-12.12% and 4.60-6.92% of the total cell number, respectively), compared with the negative control, while treatment with ECE increased the apoptotic cells (17.20-18.12% of the total cell number). The PC-12 cells treated with ECPE (25 μg/ml) or dieckol (25 and 50 μg/ml) showed apoptotic cells similar to those of the positive and untreated control groups (Fig. 3J).

### Effects of ECE, ECPE, and Dieckol on the Production of Bax, Bcl-2, and Caspase-3 Proteins in Apoptotic Pathway of PC-12 Cells

**Fig. 4:**
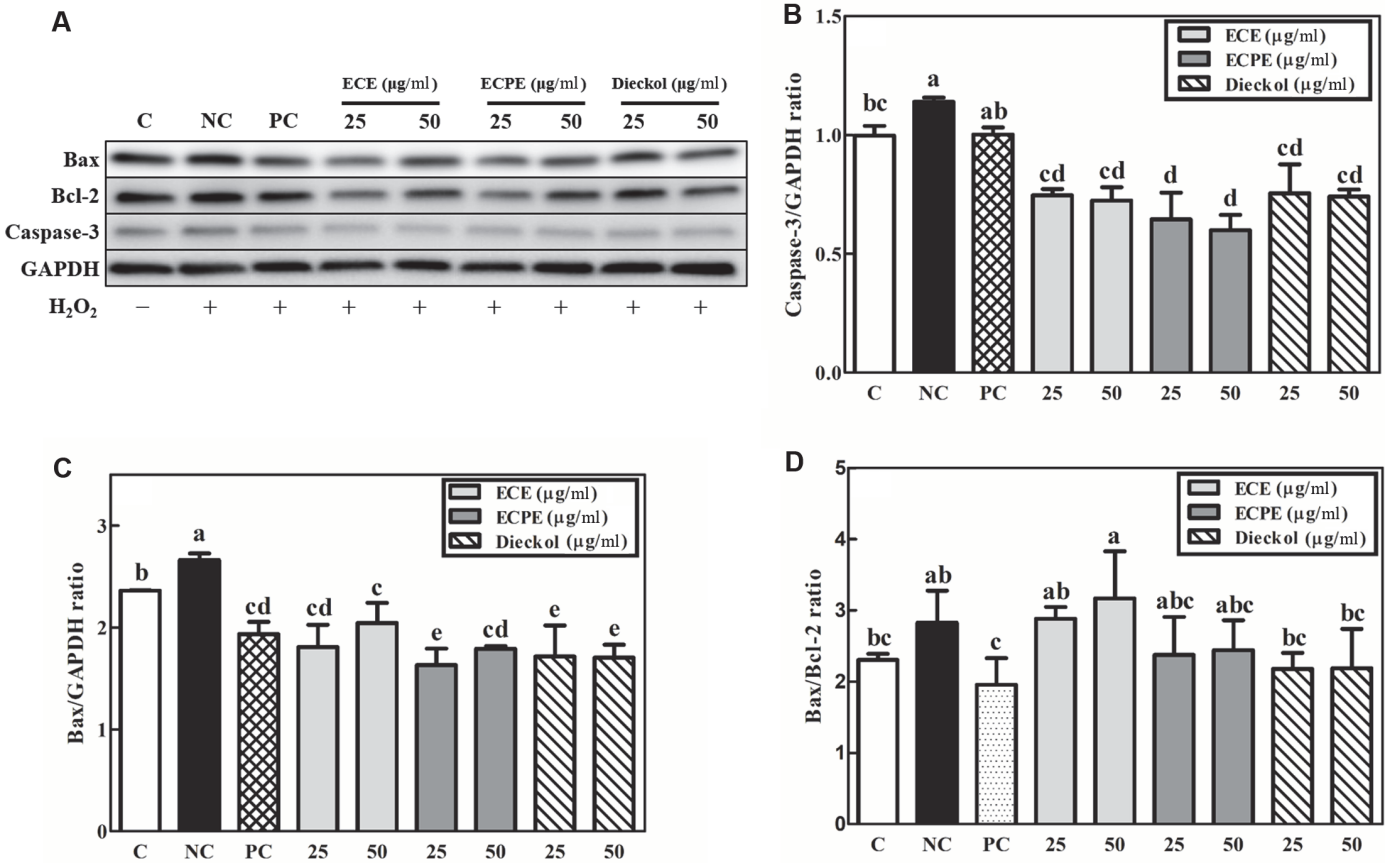
Effects of *E. cava* extract (ECE), *E. cava* phlorotannin-rich extract (ECPE), and dieckol on (A) protein production of caspase-3, Bax, Bcl-2, and GAPDH as measured by western blotting, (B) caspase-3/GAPDH ratio, (C) Bax/GAPDH ratio, and (D) Bax/Bcl-2 ratio in PC-12 cells. C, control; NC, negative control (200 μM H_2_O_2_); and PC, positive control (200 μM vitamin C). Data are displayed as the mean ± SD (bars) of three replicates. Different letters indicated a statistical difference at the *p* < 0.05 level among data groups according to Duncan’s multiple range test.

To investigate the effects of ECE, ECPE, and dieckol on the apoptotic pathway of PC-12 cells during oxidative stress, the production of three proteins (Bax, Bcl-2, and caspase-3) was qualitatively and quantitatively measured using western blot analysis (Fig. 4). Oxidative stress (200 μM of H_2_O_2_; negative control) of the PC-12 cells resulted in increased Bax and caspase-3, while treatment of H_2_O_2_-damaged cells with vitamin C (200 μM; positive control) reduced the production of the two proteins. The production of caspase-3, which is activated in apoptotic cells, decreased in the PC-12 cells treated with ECE, ECPE, or dieckol (Fig. 4B). Additionally, treatment of PC-12 cells with ECE, ECPE, or dieckol reduced the production of the pro-apoptotic protein Bax compared with the negative control (Fig. 4C). The Bax/Bcl-2 ratio of the PC-12 cells was higher under oxidative stress conditions (the negative control conditions) than in the untreated control group (Fig. 4D). Although there were no significant (*p* < 0.05) differences in the Bax/Bcl-2 ratios among the negative control, ECE, ECPE, and dieckol groups, the Bax/Bcl-2 ratios in PC-12 cells treated with ECPE or dieckol were lower than that of the negative control (Fig. 4D). PC-12 cells treated with ECPE or dieckol showed Bax/Bcl-2 ratios similar to that of the vitamin C treatment (Fig. 4D).

## Discussion

ECPE showed a 62.9% higher antioxidant capacity than ECE in the ABTS assay and a 48.3% higher antioxidant capacity than ECE in the DPPH assay. The increased antioxidant capacity of the ECPE was due to its richness in phlorotannins (Table 1), including dieckol (a hexamer of phloroglucinol), which was shown to be a major phlorotannin in both ECE and ECPE (Fig. 1). The concentration of dieckol was higher in ECPE than in ECE (Table 2), suggesting that the extraction method involving a circulating extractor and continuous separation by centrifugation used to prepare ECPE led to higher yields of phlorotannins, such as dieckol, than the general immersion method used for the ECE.

Oxidative stress-induced neuronal cell damage has been implicated in a number of diseases including neurodegenerative diseases [11]. In this study, we evaluated the neuroprotective effects of ECE, ECPE, and dieckol on H_2_O_2_-induced PC-12 cells. H_2_O_2_, one of the major ROS, easily penetrates cells and reacts with transition metal ions, such as iron and copper, producing a highly reactive hydroxyl radical (•OH) via the Fenton reaction. Therefore, the reactive H_2_O_2_ attacks the cell membrane and causes cell death [24]. Phlorotannins from *E. cava* were previously reported to increase cell viability and reduce intracellular ROS in the HT-22 murine hippocampus cell line [25]. In this study, ECE and ECPE, as well as a phlorotannin dieckol, increased the viability of neuronal cells, partly by reducing intracellular oxidative stress levels (Fig. 2). At 50 μg/ml, ECPE decreased intracellular oxidative stress more than ECE. The effectiveness of the neuronal cell protection may depend on the amounts of antioxidant phlorotannins such as dieckol in the ECE and ECPE. The increased PC-12 cell viability suggests that these two extracts protect nerve cells from oxidative stress.

The anti-apoptotic effects of the ECE and ECPE were investigated using flow cytometry. As apoptosis progresses, plasma membranes collapse. In the early apoptotic stages, phosphatidylserine, a phospholipid located on the inner plasma membrane, is exposed to the outer layers of the plasma membrane [26], where it specifically binds to annexin V-FITC. Both PI and annexin V-FITC stain cells during late apoptosis. The ECE had a less potent anti-apoptotic effect than ECPE, which concurred with the increased Bax/Bcl-2 ratio. However, ECPE (25 μg/ml) and dieckol (25 and 50 μg/ml) treatments produced anti-apoptotic effects similar to those seen in the untreated control and positive control groups (Fig. 3). The western blot and flow cytometry results in this study demonstrated the anti-apoptotic effects of the ECPE and suggest that dieckol, a major phlorotannin component of *E. cava*, influences neuronal cell apoptosis. Because apoptosis in neuronal cells is closely associated with neurodegenerative diseases, reducing apoptosis helps to prevent and treat these conditions.

The nervous system is vulnerable to oxidative stress, which can cause mitochondrial damage, ultimately leading to neuronal apoptosis [27]. Mitochondrial dysfunction induced by H_2_O_2_ promotes the activity of Bax and results in apoptosis through caspase-3 protein activation [28]. The pro-apoptotic family members Bax and Bak affect the permeability of mitochondrial outer membranes, resulting in the release of apoptogenic molecules, such as cytochrome c, and the activation of caspase in apoptosis pathways [29]. The production of Bax and caspase-3 proteins was significantly lower in cells treated with ECE, ECPE, and dieckol than those in the negative control group (Figs. 4B and 4C). The observed Bax/Bcl-2 ratios also confirmed the anti-apoptotic effects of ECE, ECPE, and dieckol (Fig. 4D). ECPE was shown to reduce the Bax/Bcl-2 ratio to such a degree that there was no significant difference from the ratio in the control (Fig. 4D). An increase in the Bax/Bcl-2 ratio at both the mRNA and protein levels promotes apoptosis through caspase-3 stimulation [30]. The discrepancy between the anti-apoptotic effects of ECE and ECPE may be ascribed to the different compositions and concentrations of phlorotannins. The results shown in Fig. 4 suggest that ECPE and dieckol are potent anti-apoptotic agents due to the presence of antioxidant phlorotannins.

In conclusion, ECPE contained higher amounts of antioxidants than ECE. The ECPE was found to have more of the bioactive dieckol and better anti-apoptotic and neuroprotective effects compared with ECE. Furthermore, the antioxidant and neuroprotective effects of the extracts varied according to the levels of bioactive phlorotannins. The neuroprotective effects of ECPE and dieckol decreased the production of pro-apoptotic proteins Bax and caspase-3, suggesting that ECPE and its main component dieckol prevent oxidative damage to neurons and inactivate their apoptotic processes. Further, in vivo animal studies and human clinical trials using phlorotannin-rich ECPE are warranted in the future. ECPE could be invaluable for the development of bioactive ingredients for improving cognitive function and memory.

## Figures and Tables

**Table 1:** Antioxidant capacity of *E. cava* extract (ECE), *E. cava* phlorotannin-rich extract (ECPE), and dieckol.

	Antioxidant capacity (mg vitamin C equivalents/g dry weight)	

	ABTS^1^	DPPH^2^
ECE	1,050.4 ± 50.2^c3^	474.6 ± 27.3^b^
ECPE	1,711.5 ± 59.2^b^	704.0 ± 75.9^a^
Dieckol	2,382.3 ± 89.3^a^	910.5 ± 69.3^a^

^1^2,2′-Azino-bis(3-ethylbenzothiazoline-6-sulphonic acid) radical scavenging assay

^2^2,2-Diphenyl-1-picrylhydrazyl radical scavenging assay

^3^Data are expressed as mean ± SD (*n* = 3). Means with different superscripts in the same column indicate significant difference by Duncan’s multiple range test (*p* < 0.05).

**Table 2:** Concentration of dieckol in *E. cava* extract (ECE) and *E. cava* phlorotannin-rich extract (ECPE).

	Concentration (mg/g)
ECE	36.97 ± 1.02^b1^
ECPE	58.99 ± 0.74^a^

^1^Data are expressed as mean ± SD (*n* = 3). Means with different superscripts in the same column indicate significant difference by Duncan’s multiple range test (*p* < 0.05).
